# Scrofula, Its Nature, Causes, and Treatment; and on the Prevention, and Eradication of the Strumous Diathesis

**Published:** 1844-07

**Authors:** 


ART. I.—*Scrofula, its Nature, Causes, and Treatment; and on the Prevention and Eradication of the Strumous Diathesis.* By W. TYLER SMITH, M.B.—*London*, 1844. 8vo, pp. 172.

IT is a great truth that there is nothing new under the sun; but although the amount ever made intelligible to us, of what was previously entirely unknown, be very small, the combinations of what is already known may be new, and may be more valuable than the products of actual discovery or invention. In the work before us we find neither new matter nor new combinations; and therefore we are constrained to say that, as it was not wanting, it should not have been published; it has contributed neither to the instruction nor to the amusement of mankind. Judging from the contents of the work itself, we must say that, though well written and evidently the production of a well-educated physician, it is the production of a man who has either not enjoyed advantages to enable him to speak with authority on the subject on which he treats, or, if he have, that most assuredly he has not profited by them. Too many books have been written on the subjects of scrofula to make it an easy task for any one to arrest attention to it; and most certainly, if this is ever accomplished, the task must be pursued in a way very different to that followed by Dr. Smith. We are, however, among those who, notwithstanding the numerous treatises on the subject, still consider it as a comparatively open field, and one which, if well cultivated, is capable of yielding abundant fruits: but the labourer must not fancy that he can reap such a harvest in his closet, even though surrounded by all the appliances that learning can minister.

We have some other faults besides those of omission wherewith to charge Dr. Smith. We do not, for instance, think it consistent with the dignity of a physician, when writing what should be a scientific work, to make it the vehicle of abuse, on insufficient grounds, of any political or economical system—even that of administering relief to the poor. It might have been well first to ascertain whether the children of the poor are, in reality, so much worse off under it, than when left to the unaided resources of their parents; whether, to use our author's own words, scrofula be "fearfully prevalent among the inmates of the present union workhouses;"—and whether "the New Poor Law be little better than a vast scheme for scrofulising the whole pauper population of Great Britain." Such statements are better suited to the columns of a newspaper than the pages of a scientific work.

We think there is not a little want of caution in expressing—as is here done—so very decided an opinion as to the ordinary curability of scrofula by medical means. If our author's opportunities of treating cases of scrofula had been many and great, we think his ideas and expressions in regard to this point would have been considerably modified. Sure we are that cases come every day into our hands, and into those of our most skilful medical friends, which drag on from year to year with only a small amount of amendment, in spite of every variety of treatment.

We think also there is some want of philosophy in the author's opinion, that the wide spread of scrofula in recent times is owing to the purification of our table salt! Really we did not conceive that so serious an evil lurked within those little baskets of salt. In our simplicity we had been accustomed fearlessly to prefer our own commodity to the browner representative of chloride of sodium used by our continental neighbours; and we confess to having heard that—in spite of the modicum of iodine said to be so important an element in the less pure compound—that scrofula is by no means an uncommon affliction in many European countries; we even fancy it has been stated that in some it is more rife than it is among ourselves. We are old enough to recollect the time when brown salt was in common use among ourselves: Where is the proof that we have more scrofula now than we had then?

In parting with the author of this book, we take the liberty of giving him one word of advice. We presume, though we know it not of certainty, that Dr. Smith is a young man; we are sure that he is not wanting in ability. His powers, however, for some years to come, will be turned to better account, for himself and for the profession, if he employs them in learning instead of teaching. There are many subjects which may be mastered without much experience, but scrofula is not among them.

